# Association between inflammation and neural plasticity biomarkers in olfactory neuroepithelium – derived cells and cognitive performance in patients with major depressive disorder

**DOI:** 10.1192/j.eurpsy.2022.1413

**Published:** 2022-09-01

**Authors:** A. Toll, M. Portella, P. Robledo, M. Barrera-Conde, R. De La Torre, J.M. Ginés, C. Diez-Aja, V. Soria, P. Lopez-Garcia, V. Pérez-Solà, P. Alvarez

**Affiliations:** 1 Parc de Salut Mar, Institut De Neuropsiquiatria I Addiccions, Barcelona, Spain; 2 Hospital de la Santa Creu i Sant Pau, Psychiatry, Barcelona, Spain; 3 Universitat Pompeu Fabra, Neuropharmacology Laboratory, Barcelona, Spain; 4 Hospital de Bellvitge, Psychiatry, Hospitalet de Llobregat, Spain

**Keywords:** biomarkers, cognition, inflammation, Depression

## Abstract

**Introduction:**

Inflammation and neural plasticity play a significant role in major depressive disorder (MDD) pathogenesis and cognitive dysfunction. The olfactory neuroepithelium (ON), closely related to the central nervous system (CNS), allows a non-invasive, low-cost study of neuropsychiatric disorders. However, few studies have used ON cells to ascertain them as biomarkers for MDD.

**Objectives:**

Determine the relationship between inflammatory/neural plasticity markers and cognitive functioning in MDD patients and healthy controls.

**Methods:**

Sample: 9 MDD patients and 7 healthy controls. Exclusion criteria: other Axis I mental disorders (patients) or any mental disorder (controls) and any inflammatory, autoimmune, or CNS diseases. Assessment: sociodemographic, clinical, and cognitive variables (CANTAB) were recorded. mRNA was isolated from ON cells and MAPK14, IL6, TNF-α, Mecp2, BDNF, GSK3, GRIA2, and FosB gene expression levels were quantified using quantitative polymerase chain reaction.

**Results:**

MDD patients showed decreased levels of BDNF (p=0.022), GSK3 (p=0.027), and working memory (p=0.024) compared with healthy controls. In healthy controls, planning was positively correlated with NRF2, BDNF, and MAPK14 gene expression. In MDD patients no correlation between cognitive parameters and inflammation/neural plasticity biomarkers was found.

**Conclusions:**

These results reveal that: (1) Plasticity biomarkers such as BDNF and GSK3 could be useful diagnostic tools for MDD (2) MDD is associated with working memory deficits; (3) no association could be determined between planning and NRF2, BDNF, and MAPK14 gene expression in MDD and (4) the ON is a promising model in the study of neuropsychiatric disorders.

**Disclosure:**

No significant relationships.

